# Evolution of Physical Self-Esteem During Pulmonary Rehabilitation in Patients with Chronic Obstructive Pulmonary Disease: An Observational Study

**DOI:** 10.3390/healthcare13010013

**Published:** 2024-12-24

**Authors:** Marc Beaumont, Arthur Mercier, Loic Péran, Anne Cécile Berriet, Catherine Le Ber, Gregory Reychler

**Affiliations:** 1 Institut National de la Santé et de la Recherche Médicale (Inserm), University de Brest, CHRU Brest, UMR 1304, Groupe d’étude de la Thrombose de Bretagne Occidentale (GETBO), 29200 Brest, France; 2 Pulmonary Rehabilitation Unit, Morlaix Hospital Centre, 29600 Morlaix, France; lperan@ch-morlaix.fr (L.P.); respi-guervenan@ch-morlaix.fr (A.C.B.); cleber@ch-morlaix.fr (C.L.B.); 3 CHRU Brest, 29200 Brest, France; mercierarthur@orange.fr; 4 Institut de Recherche Expérimentale et Clinique (IREC), Pôle de Pneumologie, ORL & Dermatologie, Université Catholique de Louvain, 1200 Brussels, Belgium; gregory.reychler@uclouvain.be; 5 Service de Pneumologie, Cliniques Universitaires Saint-Luc, 1200 Brussels, Belgium

**Keywords:** minimal clinically important difference, patient education, pulmonary disease, chronic obstructive, rehabilitation outcome, self-concept, pulmonary rehabilitation

## Abstract

**Background/Objectives**: Patients with COPD have altered self-esteem, and good self-esteem promotes personal, health, and social success. Improving self-esteem could be a method for encouraging the maintenance of physical activity. Only one study has evaluated the effects of pulmonary rehabilitation (PR) on self-esteem in moderate COPD patients. The objective was to assess the evolution of self-esteem in COPD patients of all stages of severity during PR. **Methods**: COPD patients undergoing PR were included in this prospective observational study. Patients were evaluated before and after the 4-week PR program. The objectives were to (1) assess the evolution of self-esteem using the Physical Self Inventory-6 questionnaire (PSI-6), (2) assess the evolution in each sub-score of PSI-6, (3) examine the correlations between the evolution of self-esteem and the evolution of parameters usually used during PR, and (4) determine an MID for self-esteem. **Results**: In total, 76 patients were included. We found that there was a significant increase in the total score in PSI-6 (9.29, *p* < 0.001), CI 95% [6.74; 11.83], and in the sub-scores of PSI-6. The evolution of the PSI-6 score was moderately correlated with changes in exercise capacity using STST1 (r = 0.352, *p* = 0.002) and quality of life (r = −0.361, *p* = 0.001) and weakly correlated to changes in dyspnea (r = −0.245, *p* = 0.03), anxiety (r = −0.248, *p* = 0.03), and depression (r = −0.290, *p* = 0.01). Using a distribution-based analysis, we found an MID between 5.2 and 5.6. **Conclusions**: We showed a significant increase in global score and in each sub-score of self-esteem using PSI-6 in COPD patients undergoing a PR program. We propose an MID of 5.6.

## 1. Introduction

Overall self-esteem is the evaluative dimension of the self-concept. It is defined as a conscious perception of one’s own qualities [1]. Self-esteem is multi-factorial and hierarchical and ranges from being the most global to the most specific as modeled by Fox and Corbin [2]. In this pyramid model, overall self-esteem is influenced by several core domains (professional, physical, social, etc.), with these being influenced by sub-domains. Self-esteem levels decline in old age [3], and they are negatively influenced by diseases or incapacity. Overall self-esteem is based on self-assessments of the different domains and sub-domains by the subject [4]. Thus, a person with a good perception of themselves will have good overall self-esteem.

Several studies have shown that patients with COPD have altered self-esteem or reduced well-being [5,6,7], while good self-esteem promotes personal, health, and social success [8,9,10]. Kersten [5] described changes in self-concept using a 20-item semantic meaning differential scale before and after a pulmonary rehabilitation program in 37 patients with severe COPD. The author found an altered self-concept before and an increase in self-concept after pulmonary rehabilitation. Engström et al. [6] compared the well-being of patients with COPD with matched healthy subjects. Their results showed that COPD affects a person’s functional status in most areas, particularly those daily functions that regularly require physical activity (ambulation, mobility, and home management) but also recreation/pastimes, eating, sleep/rest, and aspects of psychosocial functioning. Francis [10] reported that the use of instruments to assess self-esteem showed a positive correlation between high self-esteem and an internal locus of control, a high sociometric status, academic motivation and achievement, and high motivation to participate in sports. Conversely, high self-esteem was negatively correlated with high levels of anxiety, depression, and help-seeking behaviors. Nicolson and Anderson [7] conducted a qualitative study to describe the ways in which the disease leads to psychological distress, dependency on medication, and disruptions to social and family relationships and its impact on self-esteem. The authors reported that emotional reactions and disruption lead to reduced self-esteem.

More recently, Orth and Robbins [8] published a review of the potential benefits of self-esteem. They reported that people with high self-esteem tend to have better social relationships, tend to be more satisfied and successful at work, and tend to be healthier than those with low self-esteem. Conversely, the authors reported that individuals with low self-esteem tend to experience mental health problems, and low self-esteem is concurrently associated with antisocial behavior [8].

Improving self-esteem could promote a higher level of physical activity in these patients because some domains of self-esteem have already been related to physical activity in older adults [11]. Only one study with low-level evidence assessed the impact of pulmonary rehabilitation (PR) on self-esteem in moderate COPD patients.

The main objective of this observational study was to assess the evolution of physical self-esteem and its sub-domains during PR in COPD patients at any stage of severity. The secondary objectives were (I) to assess the relationship between the changes in self-esteem and the usual outcomes used during PR and (II) to determine an MID for the PSI-6 questionnaire.

## 2. Materials and Methods

### 2.1. Study Design

From December 2019 to September 2020, patients routinely admitted to a local 4-week PR program in the Centre Hospitalier des Pays de Morlaix (Morlaix, France) were prospectively recruited if they had moderate-to-very severe COPD diagnosed according to the American Thoracic Society/European Respiratory Society (ATS/ERS) criteria ((FEV1/FVC < 0.7; FEV1) < 80% of predictive value) at admission. The exclusion criteria were a predicted FEV1 ≥ 80%, a history of pneumonectomy or lobectomy in the last 6 months, and an inability to participate in the standard PR program or to complete the questionnaires.

The study was approved by the ethical board on 7 November 2019 (CPP-OUEST IV-Nantes, n°66/19-3, n°29BRC19.0225), complies with the Declaration of Helsinki and the current guidelines for Clinical Good Practice, and was previously registered on ClinicalTrials.gov (NCT04172155).

The research has been reported according to the STROBE statement for observational studies.

### 2.2. Instruments

The primary outcome was physical self-esteem, assessed using the Physical Self Inventory questionnaire (PSI-6). This questionnaire, developed and validated by Ninot et al. [12] in the French language [13], was adapted from the Physical Self-Perception Profile (PSPP) [2] The questionnaire consists of only six items referring to the six dimensions of hierarchical modeling of physical self-esteem: overall self-esteem, perceived physical value, physical fitness, sports competence, physical strength, and physical appearance. Each item is scored on a visual analog scale from 0 to 10 (0: not at all; 10: totally). The overall score ranges from 0 to 60, with a higher score indicating better physical self-esteem.

Complementary evaluations included clinical examination, spirometry with plethysmography, exercise capacity tests (6-Minute walk Test (6MWT), 1-Minute Sit-To-Stand Test (STST1) and cardiopulmonary exercise test), dyspnea assessments (using Dyspnea-12 questionnaire, MMRC scale, London Chest Activity of Daily Living (LCADL), Multidimensional Dyspnea profile questionnaire (MDP) (at the end of 6MWT)), isometric quadriceps maximum voluntary contraction (QMVC) and quadriceps endurance using a handheld dynamometer (MicroFET 2™, Hoggan Health biometrics, Gometz-Le-Chatel, France), inspiratory muscle strength (Micro Medical Limited PO BOX 6, Rochester, Kent, UK), health-related quality of life assessments (St. George’s Respiratory Questionnaire (SGRQ), COPD Assessment Test (CAT)), and anxiety and depression measurements (Hospital Anxiety and Depression Scale) (HADS-A and HADS-D, respectively). An educational diagnosis was also conducted.

### 2.3. Intervention

The standardized PR program was conducted 5 days per week for 4 weeks and included aerobic exercise performed on a treadmill and cycloergometer (30 min each per day), ground-based outdoor walking training, strengthening of lower and upper limb muscles groups, a therapeutic education program, aerobic gymnastics in groups, smoking cessation support, and socio-psychological and dietary advice as needed.

The outcomes were measured at admission to and at discharge from the program (except plethysmography and cardiopulmonary exercise test) by one of the three qualified physiotherapists supervising the program.

### 2.4. Statistical Analysis

In the study by Ninot et al. [12] assessing physical self-esteem in pulmonary rehabilitation, the mean score in the PSI-6 questionnaire increased from 4 to 5 per item with a standard deviation around 1.5. Assuming a 20% improvement in the overall self-esteem and in each item of PSI-6 questionnaire, with an α-error of 5% and β-error of 20%, the expected sample size was estimated to be 72 patients. In order to consider the potential exclusion of patients, 76 patients were included.

Patient characteristics were described using the appropriate descriptive parameters (mean, standard deviation, median, quartiles, frequency). For statistical tests, the law of large numbers was applied. So, the mean changes were compared using a paired Student’s *t*-test; and correlations between the different variables were analyzed using Pearson’s correlation coefficient. A *p*-value < 0.05 was considered statistically significant.

To determine the minimal important difference for PSI-6, the anchor-based and the distribution-based methods were used.

For the distribution-based method, we use the 2 standard methodologies:

- The standard deviation (SD) method: 0.5 × SD

With SD = SD pre-rehabilitation;

- And Standard Error of the Measure (SEM): SD × √1 − (test − retest)

With SD = SD pre-rehabilitation, Test–retest: ICC = 0.90 [14].

For the anchor-based method, BMI, BODE index, 6MWT, 1STST, MMRC, Dyspnea-12 questionnaire, LCADL questionnaire, CAT, SGRQ, HAD scale, quadriceps’ strength and endurance, and MIP were used as anchors. For the anchor-based estimation of the MID, we used the change from baseline in variables that correlated with the change in the D-12 score from baseline to the end of the pulmonary rehabilitation program with a correlation coefficient of at least 0.3 and a *p*-value < 0.05, as recommended [15]. Then, we used sensitivity- and specificity-based approaches with receiver operating characteristic curves to determine the best cut-off for the change in D-12 score with the established MID for the different anchors. An MID was estimated if the area under the curve exceeded 0.7 using the ROC curve [16].

## 3. Results

**Figure 1:**
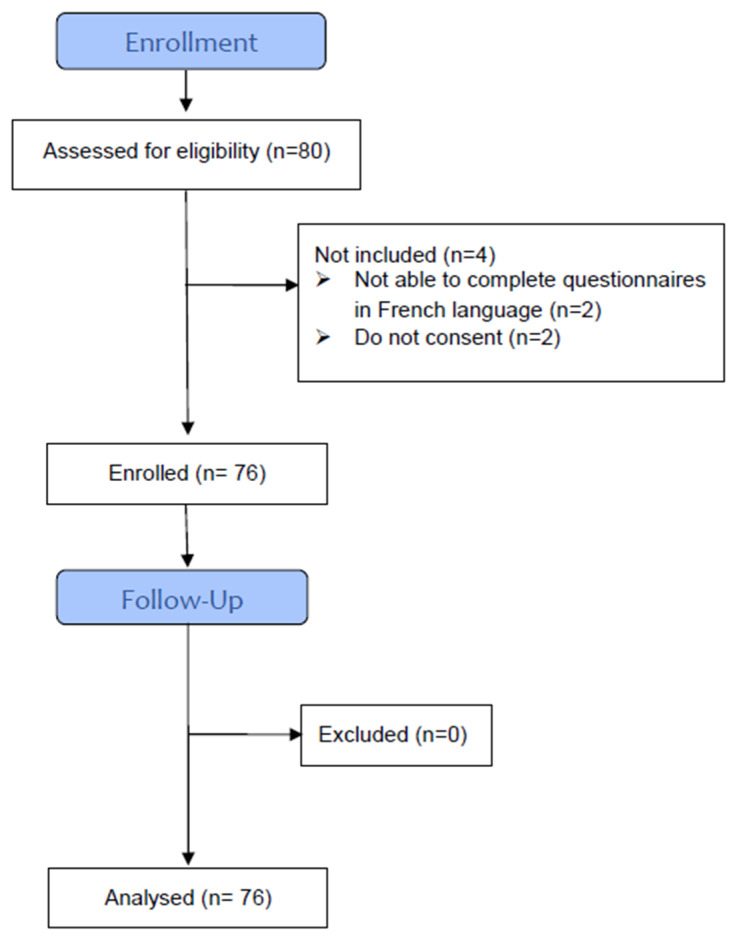
Flow chart.

Between 17 December 2019, and 29 September 2020, 80 and 76 patients were found to be eligible and ultimately included, respectively (Figure 1).

The characteristics of the analyzed patients are reported in Table 1.

### 3.1. Primary Outcome

**Figure 2:**
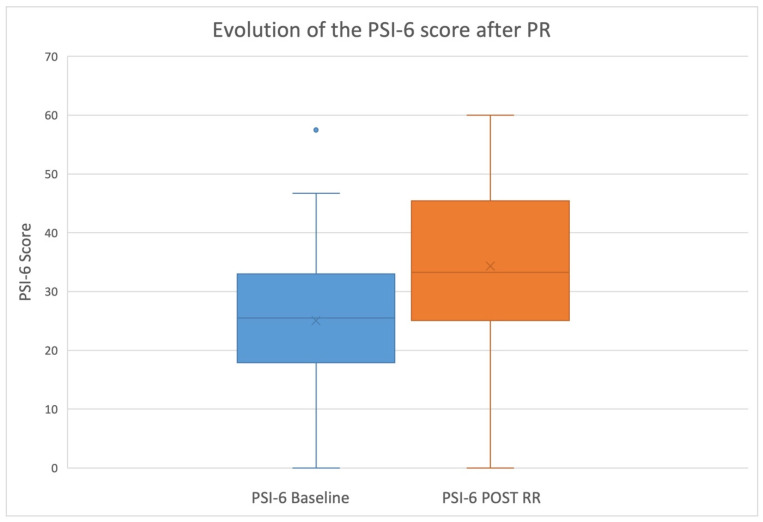
Evolution of self-esteem using PSI-6 during the PRP.

At the end of the PRP, a significant improvement in self-esteem (PSI-6) was found (9.29, *p* < 0.001) (CI 95%; 6.74; 11.83) (Figure 2).

The results for the evolution of each question in PSI-6 are reported in Table 2.

### 3.2. Secondary Outcomes

All sub scores in the PSI-6 were significantly improved: overall self-esteem, physical appearance, physical fitness, perceived physical value, physical strength, sports competence (Table 2).

The change in the PSI-6 score was moderately correlated to changes in exercise capacity measured by the STST1 (r = 0.352 *p* = 0.002) and changes quality of life using assessed by the SGRQ (r = −0.361, *p* = 0.001), and weakly correlated to changes in dyspnea assessed by the D-12 (r = −0.245, *p* = 0.03), anxiety (r = −0.248, *p* = 0.03), and depression (r = −0.290, *p* = 0.01) (Table 3).

We estimated the MID for PSI-6 as being between 5.2 and 5.6, based on the distribution method with standard error of measurement and standard deviation, respectively. (Table 4). The anchor-based method failed to determine an MID.

## 4. Discussion

The primary objective of this study was to assess the evolution of physical self-esteem in COPD patients during pulmonary rehabilitation. Secondary objectives were to assess the evolution of each sub scores for physical self-esteem, to assess the correlations between the evolution of self-esteem and the evolution of the parameters usually used during PR (exercise capacity, dyspnea, quality of life, anxiety, and depression…), and to determine an MID for the PSI-6 questionnaire.

At the end of the PRP, we observed a significant improvement in self-esteem, as measured by the PSI-6 questionnaire, and in all PSI-6 sub scores. Moderate correlations were found between improvements in self-esteem and exercise capacity (1-STST) as well as quality of life (SGRQ). Weak correlations were noted between self-esteem improvements and decreases in dyspnea, anxiety, and depression.

We estimate the MID for the PSI-6 in COPD patients as being between 5.2 and 5.6.

The baseline characteristics of our patient cohort aligned with those typically reported in pulmonary rehabilitation programs. Our findings are consistent with other studies such as that by Ninot et al. [12] and other previous studies [5,17]. Ninot et al. [12] also reported an increase in self-esteem after pulmonary rehabilitation and each dimension was similarly increased in their study. In our study, the two aspects that showed the least improvement were overall self-esteem and physical appearance. The relatively small improvements in overall self-esteem and physical appearance can be attributed to the fact that these aspects can also be influenced by other factors such as social or professional competencies [2]. So, it is not surprising that a cornerstone of pulmonary rehabilitation such as exercise training, including aerobic exercise training and strength training, leads to greater improvements in sub domains connected to physical abilities, such as perceived physical value (PPV), physical fitness (PF), sports competence (SC), and physical strength. In their study, Sonstroem and Morgan reported the positive effects of physical exercise on self-esteem and proposed a self-esteem model that includes dimensions of competence and self-acceptance [18].

This aligns with our findings about the correlation between the improvement in self-esteem and the improvements in exercise capacity (1-STST) and quality of life (SGRQ).

Several authors have compared the effects of physical activity versus no participation in physical activity on self-esteem [19,20,21,22,23,24] in populations other than COPD patients, such as older adults [19], young adults [20,24], children and adolescents [21,23], and patients with cancer [22]. In all of these populations, physical activity improves self-esteem. These results are consistent with those found in our study.

Ninot et al. [12] reported that improved perceptions of one’s physical abilities should play an important role in reducing the severity of COPD symptoms, and that a positive perception of one’s physical abilities is the primary motivational factor for continuing regular exercise training at home. These results can be used by professional caregivers to enhance the self-confidence of patients and their self-motivation for maintaining physical activity after a pulmonary rehabilitation program.

Harris and Orth [25] conducted a meta-analysis to assess the link between self-esteem and social relationships. The results of their meta-analysis showed that relationships and self-esteem reciprocally predict each other over time with similar effect sizes. The authors concluded that their findings suggest that the link between people’s social relationships and their level of self-esteem is truly reciprocal across all developmental stages, reflecting a positive feedback loop between the constructs. The authors of another meta-analysis [26] assessed the link between self-esteem and psychological problems and well-being. The authors reported that self-esteem was related to measures of well-being and psychological problems. In their conclusion, the authors invite researchers to further explore the protective role of self-esteem in the context of well-being and mental health, as well as its additive value in the treatment of people with psychological problems. A recent study [27] confirmed that the prevalence of social isolation and loneliness is high in people with COPD, and, in addition, the prevalence of psychological disorders is also well established in this population [28,29]. Thus, improving self-esteem in people with COPD could be a way to address psychological disorders and improve social relationships.

Ninot et al. [30] compared global self-esteem and physical self-scores and their stability over a three-week period in patients with COPD and healthy adults. The authors found lower mean scores for global self-esteem, physical self-worth, and each of the physical subdomains in COPD patients compared with the “healthy” group. They also observed less stability in global self-esteem, physical self-worth, and the physical subdomains in these patients. They concluded that unstable global self-esteem and physical self reflects a vulnerability to both endogenous and exogenous events. To maximize improvements in self-esteem, it might be beneficial to include sessions in the therapeutic education program that focus on enhancing overall self-esteem and identifying resources that patients can use themselves. The ‘Chinese portrait’ (a metaphorical description of oneself through a comparison with various animals, objects, places, people, characters, foods, or anything really) can be used to get to know oneself better or to discover new aspects of one’s personality, which can be used as resources for self-confidence and self-esteem. Rabiei et al. studied the effects of self-management education and support on self-efficacy, self-esteem, and quality of life among patients with epilepsy [31]. This was a quasi-experimental study: 35 patients were randomly allocated to the education group, and 35 to the control group. The Rosenberg Self-Esteem Scale was used to assess self-esteem. After the education program, the authors found a significant difference in self-esteem between the groups, favoring the education group. This study highlights the importance of therapeutic education in improving self-esteem. Although the population differs from ours, the content of the therapeutic education program appears similar to the education program typically offered during pulmonary rehabilitation. Thus, both cornerstones of pulmonary rehabilitation (exercise and therapeutic education) can improve self-esteem.

In our study, we showed a weak correlation between the improvement in self-esteem and the decrease in dyspnea, anxiety, and depression. We would expect the correlation between these outcomes to be stronger.

We hypothesized an MID between 5.2 and 5.6 points, according to the distribution-based method. The anchor-based method failed to determine an MID, because the area under the curve was <0.7. Therefore, we propose an MID of 5.6 points for the PSI-6 questionnaire after a PRP. This proposed MID must be confirmed by another prospective cohort. In our study, the patients improved their self-esteem on the PSI-6 by 9.3 (±11.1) points (*p* < 0.001), suggesting that the improvement in self-esteem was clinically significant for the patients included in this program.

Estimating the minimal important difference adds additional significance to the results and allows us to better quantify the effect we were seeking. We were able to distinguish clinical change, which is patient-based, from a significant change, which is purely statistical. Consequently, it can help the clinician to judge the relevance of integrating an intervention into their clinical routine. However, this tool is still not widely used, and estimates are rare in the scientific literature. Obtaining a new minimal important difference for the questionnaires used here in people with COPD would also allow us to refine our estimate.

### Limitations

Our study has some limitations. This is an observational study, and conducting a randomized control trial could enhance the level of evidence supporting our findings. However, it is ethically impossible to withhold pulmonary rehabilitation from patients with chronic obstructive pulmonary disease (COPD), as it is a recognized, high-level, evidenced-based, and effective treatment for their condition [32]. The variability in the PSI-6 scores was high, suggesting that there may be a confounding factor, such as the educational level of the patients. However, we did not have these data; nonetheless, no variables available in our study interacted with the PSI-6 scores. Another limitation is the duration of the program: we wonder whether the effects obtained after 4 weeks persist over time. Further studies are needed to answer this question.

Our results have several clinical implications. Firstly, self-esteem increases in individuals who engage in physical activity [19]. For patients, this can serve as a motivational resource to maintain physical activity after pulmonary rehabilitation. It is well known that sustaining an active lifestyle is one of the main objectives following a pulmonary rehabilitation program. Additionally, we propose a minimally important difference (MID) for the PSI-6, which can help care providers assess the impact of their treatment on self-esteem.

## 5. Conclusions

In our study, we showed that self-esteem was significantly improved during pulmonary rehabilitation in COPD patients. This improvement is moderately correlated with exercise capacity (1-STST) and quality of life (SGRQ). We propose an MID of 5.6 points for the PSI-6 questionnaire, but future studies are necessary to confirm our results. Assessing self-esteem during pulmonary rehabilitation is important, as it can serve as a self-motivating factor for patients to continue exercise training. In future studies, it would be interesting to assess the long-term effect of pulmonary rehabilitation on self-esteem.

## Figures and Tables

**Table 1:** Baseline characteristics of patients.

Number of patients (n)	76
Age (years)	66.3 ± 7.5
Women/Men (n) (%)	22 (29%)/54 (71%)
BMI (kg/m²)	26.3 ± 6.3
FEV1 (%pred.)	46.5 ± 17.6
FEV1/FVC	47.4 ± 11.9
RV/TLC (%pred.)	142.3 ± 34.8
BODE index	4 [2–5]
MMRC	2 [1–3]
LCADL	28.3 ± 10.4
Dyspnea-12	16.8 ± 8.8
6MWT (distance in m)	390.5 ± 119.4
6MWT (%pred)	61.8 ± 18.2
1STST (n of repetitions)	20.2 ± 6.4
MVF Quadriceps (%pred)	64.0 ± 17.8
Quadriceps Endurance (s)	44.1 ± 17.7
MIP (cmH_2_O)	70.2 ± 24.3
MIP (%pred)	80.7± 27.5
SGRQ-Total	52.1 ± 15.5
CAT	19.8 ± 6.8
HADS-D	7.2 ± 3.9
HADS-A	8.8 ± 4.2
PSI-6 total	25.0 ± 11.4
OSE	6.5 ± 2.9
PPV	4.9 ± 2.8
PF	2.8 ± 2.2
SC	2.3 ± 2.2
PA	5.2 ± 3.0
PS	3.4 ± 2.8

Values are expressed as mean ± standard deviation or median [interquartile range] or number (percentage). Abbreviations: BMI: body mass index; BODE index; B: body mass index, O: obstruction, D: dyspnea, E: exercise capacity; CAT: COPD Assessment Test; FEV1: Forced Expiratory Volume in 1 Second; HADS-A: anxiety score on the Hospital Anxiety and Depression scale; HADS-D: depression score of the Hospital Anxiety and Depression scale; LCADL: London Chest Activity Daily Living; MRC: Modified Medical Research Council dyspnea scale; MIP: Maximal Inspiratory Pressure; MVF: Maximum Voluntary Force; n: number; OSE: overall self-esteem; PA: physical appearance; PF: physical fitness; PPV: perceived physical value; PS: physical strength; PSI-6: Physical Self Inventory questionnaire; SC: sports competence; RV: Residual Volume; SGRQ: Saint-Georges Respiratory Questionnaire; TLC: Total Lung Capacity; pred: predictive value; 1STST: 1-Minute Sit-To-Stand Test; 6MWT: 6-Minute Walking Test.

**Table 2:** Evolution of self-esteem according to the PSI-6 after the PRR.

	Change in the PSI-6′ Score #	*p* Value
PSI-6 (total score)	9.29 ± 11.13	<0.001 *
OSE	0.90 ± 2.21	0.001 *
PPV	1.36 ± 2.88	<0.001 *
PF	2.08 ± 3.13	<0.001 *
SC	2.24 ± 3.22	<0.001 *
PA	0.82 ± 2.85	0.015 *
PS	1.89 ± 2.92	<0.001 *

Values are expressed as mean ± standard deviation. * *p* < 0.05. # Paired *t*-test was used. Abbreviations: PSI-6: Physical Self Inventory questionnaire; OSE: overall self-esteem; PA: physical appearance; PF: physical fitness; PPV: perceived physical value; PS: physical strength; SC: sports competence.

**Table 3:** Correlation between the evolution of the PSI-6 score and the evolution of the other evaluation criteria during the PRP.

	Correlation’s Coefficient	*p*-Value
BMI	−0.099	0.396
BODE INDEX	−0.094	0.420
6MWT	0.038	0.743
1STST	0.352	0.002 *
MMRC	−0.053	0.647
Dyspnea-12	−0.245	0.033 *
LCADL	0.076	0.514
CAT	−0.065	0.579
SGRQ-Total	−0.361	0.001 *
HAD-A	−0.248	0.032 *
HAD-D	−0.290	0.012 *
Quadricep strength	0.078	0.553
Quadricep endurance	−0.019	0.886
MIP	0.075	0.523

* *p* < 0.05 Abbreviations: BMI: body mass index; BODE index; B: body mass index, O: obstruction, D: dyspnea, E: exercise capacity; CAT: COPD Assessment Test; COPD: Chronic Obstructive Pulmonary Disease; HAD: Hospital Anxiety and Depression; LCADL: London Chest Activity Daily Living; MIP: Maximal Inspiratory Pressure; MMRC: Modified Medical Research Council; SGRQ: Saint-George’s Respiratory Questionnaire; 1STST: number of repetitions in 1-Minute Sit-To-Stand Test; 6MWT: distance in 6-Minute Walking Test.

**Table 4:** Estimation of the MID for PSI-6, according to distribution-based methods.

Method	MID Calculation	MID PSI-6
SD	0.5 × SD change	5.6
SEM	SD × √1 − (test − retest)	5.2

SD: standard deviation; SEM: standard error of the measure.

## Data Availability

Data are available on demand from mbeaumont@ch-morlaix.fr.
